# Waclaw Blaise Orlowsky and his unacknowledged discovery in neurology

**DOI:** 10.1007/s00415-017-8674-x

**Published:** 2017-11-13

**Authors:** Slawomir Gonkowski, Krystyna Makowska

**Affiliations:** Departement of Clinical Physiology, Faculty of Veterinary Medicine, University of Warmia and Mazury in Olsztyn, ul Oczapowskiego 13, 10-718 Olsztyn, Poland

**Fig. 1:**
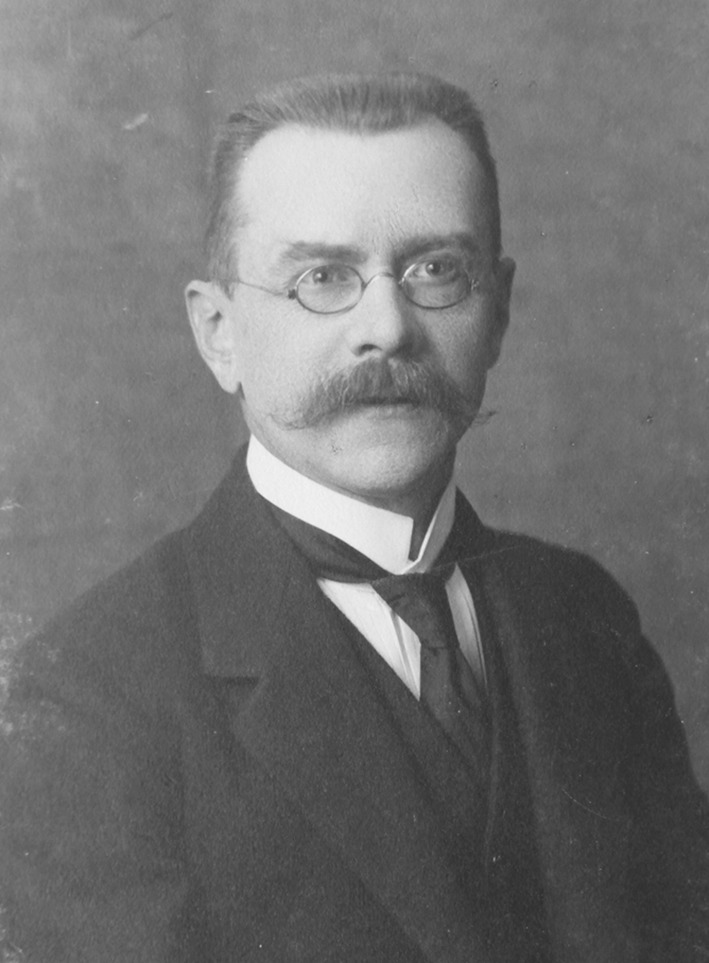
Waclaw Blaise Orlowsky. Photograph from collection of Juliusz Madalinski

Waclaw Blaise Orlowsky (Fig. 1) was born on February 2, 1868 in Brzeziny, not far from Lodz in Poland [1]. He attended elementary and middle school in Lublin. In 1888, Orlowsky went to Warsaw to study at the Faculty of Medicine in the Imperial University of Warsaw [1, 2]. During his studies, he showed interests in neurology and bacteriology. In February 1890, Orlowsky came into contact with Odo Bujwid, who was the pioneer of anti-rabies vaccination in Poland [3].

A common work of Bujwid and Orlowsky resulted in the important discovery in neurology and the histopathology of rabies. This discovery came on March 15, 1892, when Orlowski was a young, 24-year-old fourth year medical student who gave a lecture entitled “Changes in neuronal cells during rabies” during a session of the Warsaw Medical Society [1, 2]. This lecture was published in the scientific journal “Gazeta Lekarska” [4], as well as Orlowsky’s experiment was described by Bujwid in the book “Rabies in people and preventive treatment according to the method of Pasteur” [5].

Orlowsky performed his experiment on rabbits, which were infected with rabies by trepanation and injections under the dura mater with rabies germ (during the time-period of Orlowsky, scientists did not know that rabies is caused by a virus) [4]. The cervical spinal cord and medulla oblongata were collected immediately after the death of the animals (7–9 days after infection). The majority of inclusion bodies, which Orlowsky described in these words: “agleam” or “glasslike” were observed within the anterior horns of the spinal cord [4]. Unfortunately, Orlowsky did not perform studies on the brain (maybe such studies were to be performed at a later date).

Orlowski sent his results to the Pasteur Institute in Paris. Unfortunately, the studies of the young scientist were not recognized in France [1, 2]. Orlowsky never got an answer from Paris. Probably the achievement of the very young man from foreign Poland—the country under partitions—which did not even exist on the map was just ignored.

We do not know how Orlowsky reacted to the discovery of Adelchi Negri, who in 1903 described inclusion bodies in neuronal cells of cerebellum during rabies. It should be pointed out that bodies described by Negri in the brain were identical to the ones observed by Orlowski within the medulla oblongata 11 years earlier.

The fact of the matter is that Orlowsky stopped the work concerning neurological changes in rabies and saw about bacteriology. In the summer of 1892, Orlowksy went to Lublin to fight an outbreak of cholera [1]. During this stay he worked on new types of Vibrio cholerae, which later was named *Bacillus choleroides ß Orlowsky* [2].

Orlowsky got a medical degree on January 18, 1894. He went for a two-year internship, during which he worked at the Jagiellonian University in Cracow and at the University in Graz [1]. In 1896, Orlowsky came back to Warsaw and broke off a career in the sciences, what may be connected with his earlier disappointment. However, Orlowsky was still interested in rabies. In 1896, he took a job in the Warsaw Pasteur’s Center founded by Bujwid. In 1897, Orlowsky moved to Vilnius, where he opened and took on management at the Pasteur Center [1]. According to statistics carried out by Orlowsky, the Vilnius Pasteur Center between 1897 and 1939 vaccinated 15,000 persons bit by rabid dogs [2]. After the second world war, Vilnius stayed within the borders of the Soviet Union. Orlowsky moved to Olsztyn, which in 1945 was incorporated into Poland. In spite of his old age, Orlowsky tried to open a Pasteur Institute in Olsztyn, but the Polish communist authorities did not allow the project [1, 2].

Waclaw Blaise Orlowsky died in Olsztyn on October 16, 1949 at the age of 81 [1] and was buried at St. Joseph’s Cemetery. By accident and the ignorance of some people, he did not make it into the history books of neuroscience. However, it can be assumed that Orlowsky was a satisfied man. He not only became a leading specialist in the field of bacteriology, but all his life he served people who cried for help. As a pioneer of the modern anti-rabies vaccination in Poland, he saved thousands of human lives. The merits of Orlowsky have been greatly appreciated, which has found expression in the inscription on his tombstone: “Dr. Waclaw Orlowsky, one of the first Polish bacteriologists, creator of the Pasteur Institute in Vilnius, born 1868, died 1949. Let the work of his life be accounted for before the Lord”.
